# Quantifying the impact of contact tracing interview prioritisation strategies on disease transmission: A modelling study

**DOI:** 10.1371/journal.pcbi.1012906

**Published:** 2025-04-04

**Authors:** Logan Wu, Christopher M Baker, Nicholas Tierney, Kylie Carville, Jodie McVernon, Nick Golding, James M McCaw, Freya M Shearer

**Affiliations:** 1 Population Health and Immunity, Walter and Eliza Hall Institute, Melbourne, Australia; 2 School of Mathematics and Statistics, The University of Melbourne, Melbourne, Australia; 3 Melbourne Centre for Data Science, The University of Melbourne, Melbourne, Australia; 4 Centre of Excellence for Biosecurity Risk Analysis, The University of Melbourne, Melbourne, Australia; 5 The Kids Research Institute, Perth, Australia; 6 Department of Infectious Diseases, The University of Melbourne, at the Peter Doherty Institute for Infection and Immunity, Melbourne, Australia; 7 Curtin University, Perth, Australia; 8 Melbourne School of Population and Global Health, The University of Melbourne, Melbourne, Australia; Stockholms Universitet, SWEDEN

## Abstract

Contact tracing is an important public health measure used to reduce transmission of infectious diseases. Contact tracers typically conduct telephone interviews with cases to identify contacts and direct them to quarantine, with the aim of preventing onward transmission. However, in situations where caseloads exceed the capacity of the public health system, timely interviews may not be feasible for all cases. Here we present a modelling framework for assessing the impact of different case interview prioritisation strategies on disease transmission. Our model is based on Australian contact tracing procedures and informed by contact tracing data on COVID-19 cases notified in Australia from 2020 to 2021. Our results demonstrate that last-in-first-out strategies (where cases with the most recent swab or notification dates are interviewed first) are more effective at reducing transmission than first-in-first-out strategies (where cases with the oldest swab or notification dates are interviewed first) or strategies with no explicit prioritisation. To maximise the public health benefit from a given case interview capacity, public health practitioners may consider our findings when designing case interview prioritisation protocols for outbreak response.

## Author summary

Contact tracing can be an effective strategy for controlling infectious diseases, including COVID-19. Contacts of cases, identified through case interviews, may be directed to quarantine before they become infectious, interrupting chains of disease transmission. Contact tracing is limited by available resources, including the number of case interviews that can be conducted per day. When contact tracing systems have exceeded workforce capacity, it may not be possible to interview all cases in a timely manner. Hence, an approach for prioritising cases for interview is necessary to maximise transmission reduction within the available interview capacity. We use a simulation modelling framework to quantify the impact of different interview prioritisation strategies on the transmission of SARS-CoV-2. We find that prioritising the most recently identified cases for contact tracing increases the impact of test-trace-isolate-quarantine responses, since late case finding results in diminishing returns on transmission reduction. Contact tracing management systems should be designed to implement a prioritisation strategy for case interviews that achieves the greatest reduction in transmission, among other system objectives, for a given interview capacity.

## Introduction

Contact tracing is an important non-pharmaceutical public health measure used to control infectious diseases, including COVID-19. Contact tracing aims to identify individuals (“contacts”) who may have been exposed to an infection through contact with a confirmed case during their infectious period. Once contacts are identified, public health officials may then direct them to quarantine, thereby preventing onward transmission. Contact tracing is typically a manual process where public health teams conduct detailed telephone interviews with newly confirmed cases to identify contacts.

The faster that contact tracing occurs following case exposure, the more likely that chains of disease transmission will be interrupted. However, multiple points of delay exist between the exposure of a case and the identification of their contacts. These delays include but are not limited to, the time from exposure to test, test collection to test result, test result to case notification, and case notification to case interview. The case interview step can be a significant bottleneck in contact tracing processes since interviews are time-consuming, resource intensive, and limited by the number of contract tracers available [1].

When case numbers are small, as was the situation in Australia during outbreaks of COVID-19 through 2020 and early 2021, it may be possible for contact tracing teams to interview all cases on their date of notification [2]. However, when caseloads exceed the daily interview capacity, some case interviews will be delayed or missed altogether. In these situations, public health officials use various heuristics to decide which cases to interview/investigate first [1,3]. These heuristics consider factors such as the recency of case exposure and the risk of the case transmitting to others, particularly to those at risk of severe outcomes. Risk based prioritisation can be supported by information from digital pre-interview surveys of cases [3,4].

While the importance of risk factors for interview prioritisation is well recognised, an analysis of the impact of specific prioritisation strategies on disease transmission has not been conducted. Mathematical models of disease transmission and contact tracing processes have previously been used to quantify the impact of different contact tracing strategies on transmission of various pathogens [5–8]. While many studies have explored the impact of delays at various steps of the case and contact management process [8–11], case interview prioritisation strategies have received little attention. Furthermore, very few modelling studies of contact tracing have considered the limited tracing capacity of the public health system [7,12].

Here we develop a mathematical modelling framework for assessing the impact of different case interview prioritisation strategies on disease transmission when the daily interview capacity of a public health system is exceeded. Specifically, we consider a situation in the Australian state of New South Wales through 2020 when COVID-19 case numbers were very low (approximately 20 cases per day in a population of approximately eight million), with no sustained increasing or decreasing trends in caseloads [13]. In addition, test, trace, isolate and quarantine strategies were intensive, with the explicit goal of detecting all infections in chains of transmission and maintaining near-elimination status of the disease [14]. Hence while our simple modelling framework allows for random variation in the number of cases notified per day and variation in delays from swab collection to interview, it deliberately does not account for epidemic dynamics. By combining our modelling framework with contact tracing data on COVID-19 cases notified in Australia from 2020–21, we estimate the reduction in transmission of SARS-CoV-2 for five case interview prioritisation strategies: oldest swab first, newest notification first, newest swab first, newest swab first then unvaccinated first within identical swab dates, and random swab.

## Materials and methods

### Ethics statement

The study used routinely collected patient administration data from the New South Wales (NSW) Notifiable Conditions Information Management System (NCIMS). De-identified NCIMS data were securely managed to ensure patient privacy and to ensure the study’s compliance with the National Health and Medical Research Council’s Ethical Considerations in Quality Assurance and Evaluation Activities (https://www.nhmrc.gov.au/about-us/resources/ethical-considerations-quality-assurance-and-evaluation-activities). These data were provided for use in this study to support public health response under the governance of Health Protection NSW. The NSW Public Health Act (2010) allows for such release of data to identify and monitor risk factors for diseases and conditions that have a substantial adverse impact on the population and to improve service delivery. Following review, the NSW Ministry of Health determined that this study met that threshold and therefore provided approval for the study to proceed. The project oversight and approval for publication was provided by the NSW Ministry of Health.

### Overview

First, we develop a contact tracing queuing model that takes a series of cases as input. Each case carries with them attributes of relevance for our prioritisation strategies, including the relative timing of swab (in days) compared to their arrival time in the queue (i.e., the time of confirmation). We use our queuing model to generate a distribution of delays from case notification (i.e., arrival in the queue) to interview (i.e., removal from the queue), according to a specified interview prioritisation strategy.

The distribution of delays from case notification to interview under our model is then fed into a stochastic model of SARS-CoV-2 transmission and contact tracing [13], in which cases are isolated and contacts of cases (contacts identified through case interviews) are placed in quarantine. Compliance is assumed to be perfect and so those in isolation or quarantine no longer contribute to transmission. We then calculate the overall reduction in SARS-CoV-2 transmission due to isolation and quarantine for each interview prioritisation strategy. Contract tracing delays in both the queuing model and the transmission model are informed by Australian COVID-19 case data.

### Data

Line-listed data of COVID-19 cases confirmed by polymerase chain reaction (PCR) testing from 1 July 2020 to 1 February 2021 were obtained from New South Wales Health, comprising dates of case progression through the COVID-19 case management pathway. These data represent all cases notified in New South Wales during the study period, since rapid antigen self-tests were not widely available in Australia until January 2022. We used the swab date (when a test swab is registered at the point of collection) and confirmation date (when the health department is notified of a case) of each case to calculate a confirmation delay which is used as input into the queuing model. Note that we do not use the daily number of new cases from the empirical data in the queuing model, since we assume a fixed mean incoming case rate, as now described.

### Queuing model

We model the case interview process as a single server queue. Under Kendall’s notation, our system is a $D^{X}∕D^{Y}∕1$ queue [15], where notified cases enter the interview queue at deterministic (hence *D*) time intervals (daily) in batches determined by the random variable *X* and are serviced in batches determined by the random variable *Y* (also at deterministic intervals, daily). The contact tracing team is considered as a single server (*β* = 1) with a daily batch capacity *Y*.

We model the daily number of cases entering the interview queue, *X*, as a negative binomial random variable *γ* = 0 . 2, independent of time *t*, with mean (*α* = 1pace>1) and size (*r*). We model the daily number of cases interviewed (i.e., serviced), as a fixed quantity, *β* = 1, where *n* is the daily interview capacity of the public health unit (unless there are fewer than *n* cases to interview).

Because cases in the queue are prioritised for interview based on individual case attributes (e.g., swab times, notification times, vaccination status, etc.), we construct sets to represent batches of individuals arriving in the queue and being serviced. Let $\left\{x_{0},x_{1},\ldots ,x_{T}\right\}$ be a sample from *X*, with a single draw from *X* for each day from *%* to *%*. We define $A_{t}$ to be the set of cases arriving in the queue on day *t* where $|A_{t}|=x_{t}$ and $E_{t}$ to be the set of cases serviced on day *t*. Note $|E_{t}|\leq n$. Finally, we define $R_{t}$ to be the set of cases who have been in the queue for greater than *l* days on day *t* and so are no longer eligible for interview (see the Experimental setup).

With these components, we define $C_{t}$ to be the set of all cases in the queue on day *t*, which is given by $C_{t}=C_{t-1}\cup A_{t}\setminus (E_{t}\cup R_{t})$. We assume that the queue is empty prior to *%* and therefore by definition $\forall i\in \mathbb{N}\;\;C_{-i}=\emptyset$.

Each case in $A_{t}$ is assigned a day of entry to the queue $t^{\text{entry}}=t$ and a swab day $t^{\text{swab}}=t-\delta$ based on sampling a confirmation delay *%* from an empirical distribution for the delay between swabbing and notification (see the Experimental setup). This time ($t^{\text{swab}}$) is, by definition, co-incident with or prior to the time that the case enters the queue. Note that for early times *t* (strictly, times less than the maximum possible delay *%*), cases may have a negative swab time.

Cases are also assigned a vaccination status v∈ {0,1}, the outcome of an independently distributed Bernoulli trial with a probability $p_{V}$ of success (i.e., being vaccinated) (see the Experimental setup).

Finally, to capture the fact that some cases will enter the queue too late in the day to be interviewed (even if determined ‘eligible’ and so otherwise prioritised for interview), we assign each case an ‘eligible on day of entry’ status g∈ {0,1}, the outcome of an independently distributed Bernoulli trail with a probability of $p_{G}$ of success (see the Experimental setup).

To determine which cases are serviced, we construct the set $P_{t}=C_{t-1}\;\cup \;A_{t}^{\prime }$, where $A_{t}^{\prime }:=\left\{a\;\;\in \;\;A_{t}:\;\;a\;\;\text{is}\;\;\text{eligible}\;\;\text{for}\;\;\text{processing}\;\;\text{given}\;\;\text{by}\;\;\text{its}\;\;\text{attribute}\;\;g\;\;\text{having}\;\;\text{value}\;\;1\right\}$ includes only those cases entering the queue on day *t* who are also eligible for processing on that day. Early on, before the queue is “full" or more precisely $|P_{t}|\leq n$ then all eligible cases are interviewed and $E_{t}=P_{t}$.

If the queue is sufficiently large (determined by $\left|C_{t}\right|$) such that processing capacity is exceeded (as it will be except for at initialisation), $E_{t}$ is constructed as the *n* highest-priority cases in $P_{t}$ ranked by a given prioritisation strategy.

We consider four prioritisation strategies: random swab; oldest swab first; newest notification first; newest swab first; and newest swab first, then unvaccinated first within identical swab dates (see the Experimental setup). The set $P_{t}$ is sorted according to the specified prioritisation strategy and the first *n* elements of $P_{t}$ are used to construct $E_{t}$.

By construction, our queue is a Markov Chain. To generate a distribution of delays from case notification to interview, we simulate the queue forward from *%*, drawing $N_{\text{samples}}$ from the stationary distribution of the chain. We choose $N_{\text{samples}}$ sufficiently large such that the Monte Carlo error for the mean and standard error of our computed quantities of interest are negligible. Given that the chain is empty for *%*, and that cases entering the queue just prior to the end of the forward simulation are not necessarily eligible (yet) to be processed for interview, we discard sufficient samples from both the beginning and the end of the chain. These burn-in and burn-out periods are very short in practice given the empirical distributions governing the simulation.

**Fig 1:**
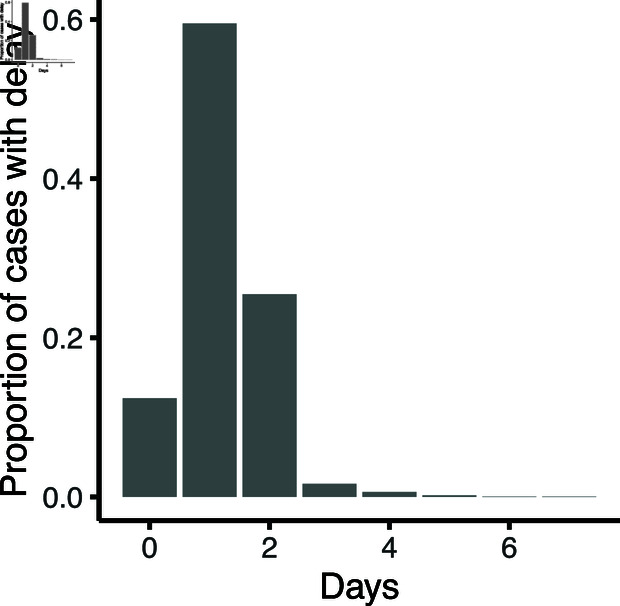
Empirical distribution of delays from swab collection to case notification from COVID-19 cases notified in the Australian state of New South Wales between 1 July 2020 and 1 February 2021.

The final outputs from the queuing model is a distribution of delays from swab to notification, drawn from the empirical distribution, and a distribution of delays from notification to interview, generated by the model.

### Experimental setup

For the primary analysis, we fix the mean daily rate for batch size, *X*, to 20 cases per day (and the overdispersion parameter r=10), broadly reflecting the daily number of COVID-19 cases notified in New South Wales from July 2020 to February 2021. We also examine two other values for the dispersion parameter (*r*), representing high (*%*) and negligible (r=100) overdispersion in the arrival rate (S1 Fig). The input confirmation delay for each case, *%*, is drawn from empirical observations (Fig 1). We assume that 45% of incoming cases are vaccinated (i.e., we set $p_{V}$ to 0.45) which is consistent with the population-level two-dose vaccination coverage in New South Wales (approximately 60%) and global understanding of vaccine protection against asymptomatic and symptomatic infection with the Delta SARS-CoV-2 variant at the time of analysis [13]. Any cases not interviewed within five days of notification are removed from the queue and hence never interviewed (i.e., we set *l* to 5) because extremely late interviews have little effect on transmission reduction [16]. Finally, we assume that 20% of case notifications arriving on a given day cannot be interviewed on the same day (i.e., we set $p_{G}$ to 0.8) due to a range of reasons such as out-of-hours notification (i.e., the interviewer or interviewee is not available) or missing contact details, even if the queue is empty. This value of 20% is based on the authors’ experience with an Australian state department of health’s COVID-19 notification system.

We explore five interview prioritisation strategies using the queuing model and feed outputs—the distribution of delays from case notification to interview—for each strategy into the transmission model to compute the overall reduction in transmission due to contact tracing

1. Random swab
2. Oldest swab first
3. Newest notification first
4. Newest swab first
5. Newest swab first, then unvaccinated first within identical swab dates

We assess each prioritisation strategy under 20% (*%*), 50% (n=10), and 80% (n=16) daily interview capacities. For example, under 20% capacity, the interview workforce is able to interview up to 20% of the mean number of incoming cases per day. We also examine the queuing model outputs (i.e., distribution of delays from swab to case notification) for a range of daily mean incoming case rates where the daily interview capacity (*n*) is a fixed proportion of the mean incoming case rate (*X*) (S2 Fig).

#### Estimating the reduction in transmission

We estimate the reduction in SARS-CoV-2 transmission under different prioritisation strategies by passing the resulting delay distributions from the queuing model into a stochastic simulation model of SARS-CoV-2 transmission and contact tracing developed by Shearer and colleagues [13]. The stochastic simulation model is described in detail in Shearer et al. [13]. Briefly, the model represents the relationship between contact tracing delays, symptomatic detection, and times from infection to isolation in successive chains of contact tracing. The model accounts for two modes of case detection: active detection by downstream contact tracing from the case’s infector, and passive detection by the case developing symptoms and seeking a test. By repeatedly sampling from distributions representing these processes via a recursive sampling algorithm, the model generates distributions of the time from infection to isolation for cases as described in Eqs 6–13 of Shearer et al. [13]. The distribution of times from infection to isolation are then directly translated into reductions in potential for onward transmission by following the process described by Eqs 1 and 2 of [13] which are re-described here for convenience.

We calculate the expected reduction in transmission for detected infections (*s*; a multiplier on the reproduction number) due to isolation as the finite sum (up to a maximum of M=20 days) of the product of the cumulative distribution function of the isolation delay distribution ($F_{\pi_{I}}(x)=\sum_{x^{\prime }=0}^{x}\pi_{I}(x^{\prime })$, where $\pi_{I}$ is the probability mass function of the discrete distribution of delays from infection to isolation) and the probability mass function of the generation interval distribution in the absence of isolation ($\pi_{G}$, delay from infection to onward transmission for individuals not in isolation):


$$\begin{array}{ccc} s=\overset{M}{\underset{\kappa =0}{\sum }}F_{\pi_{I}}(\kappa )\pi_{G}(\kappa ) & & \end{array}$$ (1)

Since the data are only available on a daily time step, we model both $\pi_{I}$ and $\pi_{G}$ as discrete probability distributions over times since infection to isolation. The generation interval distribution is modelled as a discretised log-normal, with parameters given by posterior mean estimates from [17] (with parameters μ=1.376 and σ=0.567 giving a distribution of mean of 4.7 days and standard deviation of 3.0 days):


$$\begin{array}{ccc} \pi_{G}(\kappa |\mu ,\sigma )=\int_{\kappa }^{\kappa +1}\text{log-normal}(z|\mu ,\sigma )\;\text{d}z & & \end{array}$$ (2)

The stochastic simulation model of [13] takes a number of component distributions as input, including the contact tracing delay (referred to as $T_{C}$ in [13]). The variable $T_{C}$ comprises several sub-component distributions including: the time from swab collection to case confirmation; the time from case confirmation to case interview; and the time from interview to contact notification and swab collection. Here we supplied the model with a distribution of times for the first two of these: time from swab collection to case confirmation, as drawn from the empirical distribution, and time from case confirmation to case interview for each interview prioritisation strategy, as generated by the queuing model. The times from case interview to contact notification and swab collection were as estimated by Shearer et al. [13].

For our study, the transmission model assumes that vaccinated cases are 36% less infectious than unvaccinated cases (i.e., a 36% reduction in contagiousness given breakthrough infection). This value is based on estimates of vaccine effectiveness against onward transmission in breakthrough infections of the Delta variant of SARS-CoV-2 following two doses of the AstraZeneca (ChAdOx1 nCoV-19) vaccine as reported by Eyre and colleagues in the first version of a pre-print published in 2021 [18]. While this estimate was later updated in the peer-reviewed publication in 2022 [19], we use this value as our study was conducted prior to the final publication date in 2022 as part of a suite of research to support Australian government decision-making on COVID-19 [20]. It should also be noted that vaccine effectiveness against onward transmission in breakthrough infections is expected to be lower for Omicron variants compared to Delta [21].

The transmission model implicitly assumes that all infections are (eventually) reported as cases. In the hypothetical scenario that all cases were identified and isolated instantly after infection, this would result in a 100% reduction in transmission. However, at low levels of disease prevalence (as seen in New South Wales during the study period), these reductions scale linearly with the rate of case ascertainment. For example, if instead only 30% of infections were detected, all of these transmission reductions would be scaled by 30%. This therefore does not influence the relative benefits of the different case interview prioritisation strategies considered here.

## Results

**Fig 2:**
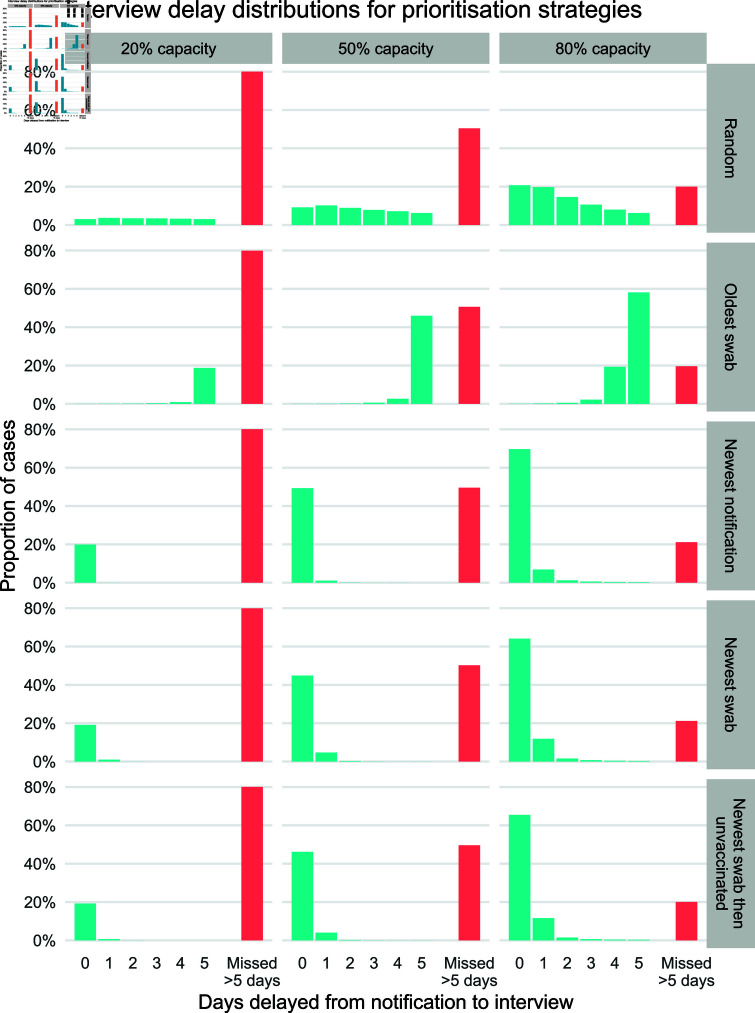
Interview delay distributions for twelve combinations of different prioritisation strategies and interview capacities. A delay of zero days means that cases were interviewed on the same day their notification was confirmed by the health authority. If the delay exceeds five days, the interview is missed.

Running our queueing simulation under different interview prioritisation strategies generates outputs of the steady-state delay distributions; i.e., a sample population of the delays experienced by a case. The different resulting distributions are shown in Fig 2. The number of missed interviews directly corresponds to the interview capacity, with the number of missed interviews increasing as capacity decreases. The successful interviews are distributed from zero to five days, and the shape of these distributions depends on the prioritisation strategy. ‘Newest’ strategies exhibit shorter delays to case interviews on average compared to ‘random’ and ‘oldest’ strategies.

In S2 Fig, we demonstrate that the delay distributions from the queuing model depend only on the ratio of mean arrival rate to interview capacity, rather than the absolute value of either of these quantities. For example, for a case entering a system with 50% mean capacity, the probability of a given delay is the same irrespective of whether 10 or 1 000 cases will arrive that day (given 5 or 500 interviews respectively).

**Fig 3:**
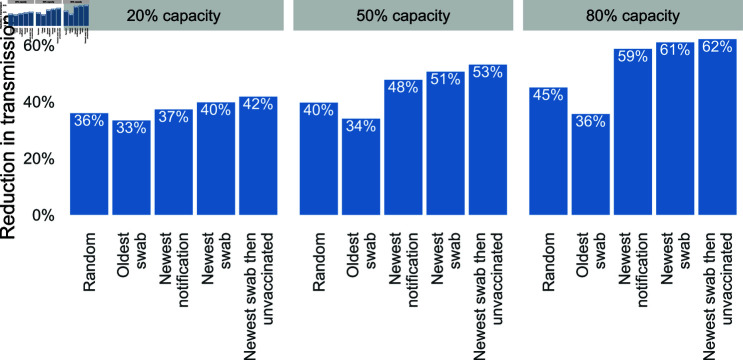
Estimated reduction in transmission for different combinations of interview prioritisation strategy and interview capacity as a percentage of the mean incoming case number. The reduction is the overall effect of the test-trace-isolate-quarantine system, where we assume 100% compliance with isolation and quarantine.

The delay distributions shown in Fig 2 were fed into the transmission model to estimate the overall reduction in transmission due to contact tracing, under each case interview prioritisation strategy. Outputs are shown in Fig 3 where the overall transmission reduction scales between zero (no change) and 100% (complete prevention of transmission).

We find that the two strategies prioritising the newest swab first provide the greatest reduction in transmission, followed by the newest notification first, random swabs, and finally the oldest swab first strategy. Furthermore, additional prioritisation by unvaccinated status when ordering cases swabbed on the same day provides a modest benefit in transmission reduction as vaccinated cases contribute less to downstream transmission in our transmission model.

The ranking of strategies is consistent within a given workforce capacity, but the absolute and relative performance depends on the capacity. As capacity increases from 20% to 50% to 80%, the reduction in transmission increases from around 40% to as high as 60%. Furthermore, the difference in transmission reduction between oldest swab (the poorest performing strategy) and all other strategies increases as capacity increases. That is, the greater the workforce capacity, the greater the potential gain (or loss) or gain from an effective (or ineffective) strategy. For example, the most effective strategy (newest swab) at 20% capacity is almost as effective as interviewing people at random in the queue at 80% workforce capacity, highlighting how an effective strategy can compensate for having far fewer interviewers. The least effective strategy (oldest swab) receives no benefit from additional capacity because the extra capacity is used to conduct interviews on the backlog of case notifications which provides little benefit in terms of transmission reduction. Our results are consistent irrespective of our choice of the overdispersion parameter for the incoming case rate (S1 Fig).

## Discussion

We developed a mathematical modelling framework for assessing the impact of different case interview prioritisation strategies on disease transmission and applied it to COVID-19 case data collected in Australia from 2020 to 2021. We investigated five different case interview prioritisation strategies: oldest swab first, newest notification first, newest swab first, newest swab first then unvaccinated first within identical swab dates, and random swab.

We found that last-in-first-out strategies (newest notification first, newest swab first, and newest swab first then unvaccinated first) are more effective at reducing transmission than a first-in-first-out strategy (oldest swab first) or no explicit prioritisation (random swab). These results support an overarching principle–—the case who should be interviewed next is the one where contact tracing has the potential to avert the greatest number of downstream infections. The strategy that performs best (the newest swab first, and the variation of prioritising vaccinated people too) deprioritises those who are expected to have already contributed to the bulk of their downstream infections and, therefore, have reduced opportunity to prevent infections via contact tracing. Our results also demonstrate that the benefits of last-in-first-out strategies increase as daily interview capacity increases, i.e., the most effective strategies confer the greatest absolute and relative benefit when the daily interview capacity is highest.

During the COVID-19 pandemic, many countries employed smartphone-based digital contact tracing to augment manual processes, since faster and more complete contact identification is theoretically possible compared to using case interviews [22]. However, implementation issues, including poor uptake of tracing applications by smartphone users, have largely prevented them from contributing meaningfully to disease control [23]. Hence studies that aim to improve manual contact tracing processes, such as ours, remain important.

A strength of our approach is in the use of data on actual contact tracing delays as input, which enabled us to generate results that were meaningful for a specific disease, population, and public health system context. Specifically, our results relate to situations of relatively stable COVID-19 epidemic dynamics, as seen in the Australia state of New South Wales in 2020. Our approach deliberately does not consider the feedback effects of changes in incoming caseloads on the contact tracing effectiveness of the public health unit, or vice versa. Extending our approach to incorporate epidemic dynamics is an important avenue for future research and would require a model framework where the interview capacity is fixed but case numbers may fluctuate through time, with interdependent case dynamics and contact tracing processes. While the absolute values of transmission reduction estimated in our study would not generalise to situations with less stable epidemic dynamics, our results at different fixed capacities suggest that the ranking of strategies (and the diminishing differences in transmission reduction between strategies as the proportion of daily incoming cases interviewed decreased) would still emerge from a full dynamical investigation.

Following the adoption of a national ‘re-opening’ plan in July 2021, findings from our research were reported to key SARS-CoV-2 decision-making committees in Australia in November 2021 [24] as part of a wider package of work to inform policy changes as SARS-CoV-2 became established in the broader population. However, to apply our findings in practice, contact tracing teams would require support from digital infrastructure. A manually maintained spreadsheet is unlikely to be sufficient; at the very least, a programmed spreadsheet or dedicated platform is required to dynamically allocate the highest priority interview to contact tracers as each interview finishes and a new tracer becomes available.

Previous mathematical modelling studies have demonstrated how reducing delays to contact tracing and/or increasing the fraction of contacts traced can reduce transmission of SARS-CoV-2 [7,9,11]. However, very few modelling studies of contact tracing processes have considered the limited capacity of the public health system [7,12], and none have explored this in the context of case interview prioritisation. Kaplan and colleagues developed a model of smallpox transmission and contact tracing which, like our study, includes a tracing queue (specifically a queuing compartment in their ordinary differential equation model) where the rate at which individuals exit the queue depends upon the number of contact tracers available. However, queue exit times are not governed by an individual’s arrival time (since this was not necessary to fulfil their study aims), and so would be equivalent to our random swab strategy [12]. Meister and Kleinberg developed an algorithm for determining the optimal ordering for tracing of identified contacts, according to each contact’s probability of infection, recency of exposure, and the number of other contacts they may have exposed [25]. However, they do not model the impact of contact tracing on transmission as they only consider the direct benefits of timely medical treatment due to contact notification. Indirect clinical benefits arising from the prevention of onward transmission via contact tracing and quarantine are therefore not considered.

Our study has several limitations. Since case data were provided at a daily resolution, it was necessary to process simulations in daily batches so that empirical delays could be incorporated into our model without making intra-day assumptions. While this is more computationally efficient than sub-daily processing, if data were available at a sub-daily resolution (e.g., with date stamps), it would be possible to explore additional processing considerations such as the relative merits of batch processing versus online processing (where the queue order would be updated in real-time in response to newly notified cases). For pathogens such as SARS-CoV-2 where the contact tracing window is relatively short and small reductions in delays can lead to significant impacts on disease control [9], shorter-than-daily batch processing or online processing may result in further reductions in transmission.

Our study does not consider risk factors for onward transmission except for the vaccination status of cases (since we assume that unvaccinated cases are more infectious than vaccinated cases). Other factors such as case occupation, age, or housing type, or whether a case visited or worked in a high-risk location/setting during their infectious period, are often incorporated in protocols for case interview prioritisation [1,4,16,26]. For example, contact tracers may prioritise interviewing a person who works in a healthcare setting over an academic working from home, since the healthcare worker likely has many more in-person interactions each day than the academic. In many countries, interviews of COVID-19 cases linked to high-priority settings such as aged care were expedited by health departments [16,26]. A more comprehensive risk prioritisation model would assume the existence of pre-interview case surveys, potentially conducted at the time of swab collection, so that information on key risk factors is available to a prioritisation algorithm as soon as newly confirmed cases enter the interview queue. The relationship between these variables and onward transmission would also need to be quantified to make full use of them within a prioritisation algorithm. Nonetheless, if these additional variables were incorporated in a prioritisation algorithm, we expect that our overarching findings would hold, because any delay to interview for these ‘high risk’ cases would result in less benefit in terms of disease control. Additionally, our prioritisation algorithm only uses vaccination as a ‘tiebreaker’ for cases with the same swab/notification date. Other prioritisation strategies could be explored where the relative onward transmission risk for cases exhibiting different combinations of risk factors is considered. Finally, our algorithm only considers the prioritisation of cases according to attributes that increase the risk of onward transmission but contact tracing may have objectives other than transmission reduction, such as the early treatment of infected contacts. The algorithm presented here could be extended to consider multiple (potentially conflicting) contact tracing objectives. Multi-objective problems may carry additional challenges, such as ethical considerations in prioritising the tracing of some cases over others.

Our findings can inform outbreak response for COVID-19 and other diseases with similar biological characteristics affecting transmission. Contact tracing is time-sensitive for any disease that spreads slowly enough to enable a tracing window but sufficiently fast that even small reductions in tracing delays can have a large benefit for outbreak control [5]. While the exact numerical results provided here will vary depending upon the infectious disease and the population in which the disease is spreading, our modelling framework can be applied to assess the impact of interview prioritisation strategies using contact tracing data collected in other contexts. Furthermore, our approach is applicable to other case and contact management processes that are both time-sensitive and where public health workloads may exceed the system capacity, including diagnostic testing procedures.

## Supporting information

S1 FigEstimated reduction in transmission for different combinations of interview prioritisation strategy and workforce capacity as a percentage of the mean incoming case number (*X*) and three different values for the dispersion (size) parameter (*r*), representing high (*%*), moderate (r=10), and negligible (r=100) overdispersion in the arrival rate. The reduction is the overall effect of the test-trace-isolate-quarantine system, where we assume 100% compliance with isolation and quarantine.(EPS)

S2 Fig Interview delay distributions (i.e., queuing model outputs) under the ‘random’ interview prioritisation strategy exhibit similar mean values (dots) for a range of mean arrival rates where the interview capacity (20%, 50%, or 80%) is a fixed proportion of the mean arrival rate of cases into the queue. Intervals show variation of the distribution after simulating 1 000 days of interviews, represented as a 95% Wilson CI of the mean proportions.
(EPS)
